# The Impact of Optimized Blinking on Vision and Related Parameters in Individuals With Computer Vision Syndrome: A Single-Blind Randomized Controlled Trial

**DOI:** 10.7759/cureus.67653

**Published:** 2024-08-24

**Authors:** Parul Sadhwani, Lalitha CS, Shovna Dash, Soumyakanta Mohanty

**Affiliations:** 1 Ophthalmology, Kalinga Institute of Medical Sciences, Bhubaneswar, IND

**Keywords:** near point of accommodation, visual acuity, tear film breakup time, blinking, computer vision syndrome (cvs)

## Abstract

Introduction: Computer vision syndrome (CVS) has become a significant issue for individuals working on computers and digital devices for extended periods. The ocular and periocular symptoms and signs associated with CVS are a major concern, affecting individuals physically and financially. Additionally, CVS has been linked to the rapid progression of myopia, exacerbating the situation. Blinking has been one of the major treatment methods for the treatment of CVS. This study presents a unique and novel randomized controlled therapeutic trial that evaluates the impact of extended blinking therapy on eye health and vision, along with other related parameters.

Materials and methods: The present study is a randomized controlled trial conducted from September 2022 to April 2024. Participants aged 18-40 with CVS and a computer vision syndrome questionnaire (CVS-Q) score of ≥6, with mild to moderate refractive error (between -6D and +4D), were included. The sample size was determined based on a pilot study, resulting in a minimum required sample size of 36 patients (18 cases and 18 controls). Participants were randomly assigned to either the case (interventional) or control (conventional) group and were followed up for six months. Cases received conventional CVS treatment plus optimized blinking exercises, while controls received conventional therapy only. Comprehensive ocular assessments were conducted bi-monthly over six months, evaluating changes in uncorrected visual acuity (UCVA), refractive error, near point of accommodation (NPA), near point of convergence (NPC), Schirmer’s test, and tear film breakup time (TBUT).

Results: The study included 20 patients in the case group and 18 in the control group, primarily aged 20-29 (60.5%). Most patients used laptops for their activities (55.26%). The CVS-Q score significantly decreased in both groups following treatment, with both cases and controls showing significant improvement (p<0.001 for both groups). UCVA in the right eye (RE) and left eye (LE) of the cases improved significantly post-treatment in the interventional group (RE: p=0.002; LE: p<0.001). A significant change in refractive error, which is measured as spherical equivalent (SE), was seen among cases following treatment (RE: p<0.001; LE: p=0.021). Controls showed no significant changes in visual acuity or refractive error. The NPA in the cases improved significantly in the RE (p=0.027) but not in the left. The NPC in the intervention group showed no significant change, while controls showed considerable improvement (p=0.042). Schirmer's test results showed no significant change in either group. However, TBUT in the cases improved significantly (RE: p<0.001; LE: p<0.001). In the controls, TBUT decreased significantly, indicating a deterioration in tear film stability. Asthenopia grades improved considerably in cases, while controls showed only some improvement. Severe symptoms still remained in the control group, emphasizing the potential benefits of the blinking exercise in reducing asthenopia symptoms.

Conclusion: Optimized blinking therapy significantly improves vision and refractive error, tear film stability, and discomfort, making it beneficial for chronic computer users to maintain ocular health and enhance productivity and quality of life.

## Introduction

Computer vision syndrome (CVS), also called digital eye strain (DES), describes a group of eye and vision-related problems resulting from prolonged use of computers, tablets, e-readers, and cell phones [1]. India’s digital population is approximately 624 million active users as of February 2021, with approximately 8.5 hours per day spent viewing electronic screens [2]. Different review article studies found that 64-90% of computer users experience visual symptoms, including eyestrain, headaches, ocular discomfort, dry eye, diplopia, and blurred vision near or when looking into the distance after prolonged computer use [3].

In a decade or two, 50% of the world's population is going to be myopic and a greater number shall be highly myopic as stated by WHO, one of the single most important causes being increased screen time [4,5]. Various methods have been tried to address this concern, with the use of anti-reflective protective glasses/screens, lubrication, the 20-20-20 rule, and some modifications in workspace ergonomics at the forefront [1].

Dry eyes have been a major component of CVS, attributed to altered blinking patterns [6]. Blinking frequency and completeness are reduced during digital screen exposure, compromising meibomian secretion and distribution, causing tear film instability and dry eyes [7]. Proper blinking plays a vital role in dry eye management. It also helps in the genesis of a new tear film, thus replenishing evaporated aqueous tears and wetting the ocular surface. Dry ocular surface-associated blurring also dissipates with proper blinking. Therefore, maintaining ocular hygiene, tear film dynamics, and proper screen use behavior can prevent many symptoms of CVS [8].

At this juncture of the CVS boom, we have assumed that dry eye-associated visual blurring may stimulate the onset or progression of refractive changes seen with digital eyestrain. Blinking at an appropriate rate, interval, and completeness can improve the tear film profile and may contribute to improving refractive error and other related parameters. Therefore, we aim to study the effect of voluntary optimization of blinking on visual parameters in CVS. To assess the effectiveness of blinking optimization, changes in various parameters of CVS, along with changes in tear film profile and visual parameters before and after treatment among the groups, were the aims and objectives of our study. This could help improve work efficiency, decrease treatment costs, and combat this public health problem.

## Materials and methods

Study details

This randomized controlled trial was conducted from September 2022 to April 2024 in the Department of Ophthalmology, Pradyumna Bal Memorial Hospital, KIMS, Bhubaneswar, with registration no: CTRI/2023/10/059100. Participants meeting the inclusion criteria were randomly assigned to case or control groups after obtaining ethics approval from the Institute Ethics Committee (KIIT/KIMS/IEC/953/2022) and were followed up for six months. The study population mainly consisted of individuals from the surrounding IT hub, schools, and colleges, making participant recruitment convenient after obtaining informed consent.

Sample size

The lack of literature about the effect of blinking exercises on vision and refractive correction prompted us to undertake a pilot study by taking 42 convenient samples, i.e., 21 cases and 21 controls. In the pilot study, the average change in the spherical equivalent (SE) in the right eye (RE) of cases (intervention group) was -0.237 with SD±0.32, and the average change in SE of the RE for controls was 0.145 with SD±0.26, which was statistically significant (p-value<0.05). At a 5% level of significance and a 95% confidence interval, the minimum required sample size for each group was 18 patients, i.e., a total of 36 patients.

Inclusion criteria were patients aged 18-40 with CVS, with or without mild to moderate refractive error (below -6D and below +4D). Exclusion criteria exclude females during their menstrual period, drug history was taken, and history of pregnancy was taken for every female participant. Other ocular diseases causing dry eyes, systemic illnesses like uncontrolled diabetes or thyroid disease, secondary Sjogren’s syndrome, use of ocular medications causing dry eyes, history of ocular surgery, or use of contact lenses were also excluded.

Study procedure

The demographic profiles of all eligible patients were included in the study. A detailed clinical history, including the type of digital device used, duration of use, associated symptoms, and detailed ocular and systemic history, was taken. CVS-Q questionnaires were used to assess CVS scores and to grade the asthenopic symptoms. An initial systemic general examination was done. A detailed ocular examination was performed along with a few specific tests before and after six months of treatment.

The study participants were randomly allocated into two groups (i.e., cases and controls) for treatment purposes, with 18 patients in each group. This was done by a computer-generated random numbering system with only the patients blinded (single-blinded study). Treatment was given to both groups. Group 1 received conventional CVS treatment and optimized blinking, while Group 2 received only conventional treatment.

**Figure 1:**
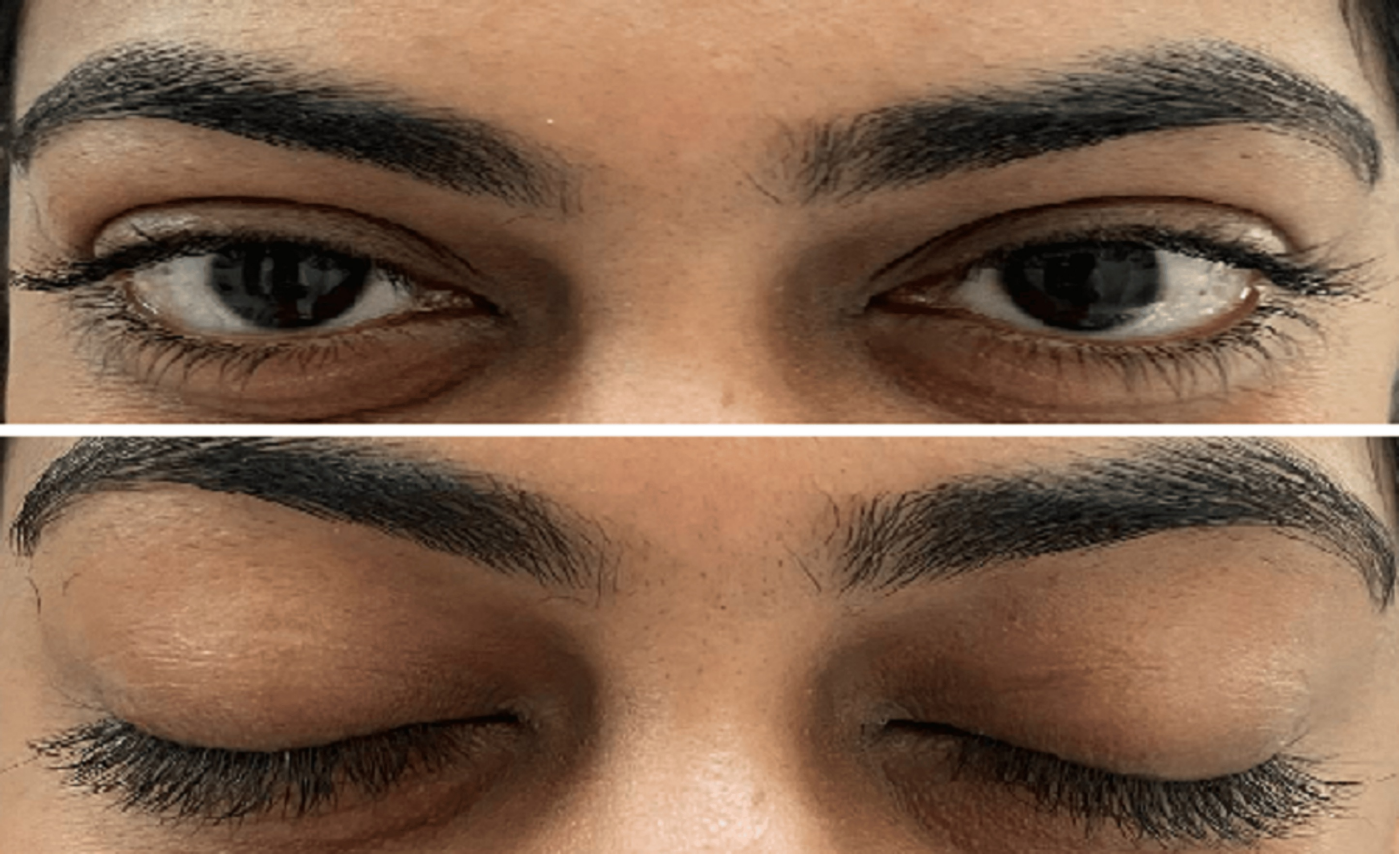
Patient looks at a distance of 6 meters or more for 5 sec (A) followed by complete closure of eyelids for 5 sec (B) and following this blinking exercise cycle for five minutes

The conventional treatment included the 20-20-20 rule: asking the patient to look at an object 20 feet away for 20 sec every 20 minutes, blue light-blocking glasses, followed by lubricating eye drops. In the blinking exercise group, the patient looks at a distance of 6 meters or more for 5 sec (Figure 1A), followed by complete closure of eyelids for 5 sec (Figure 1B), and follows this blinking exercise cycle for five minutes, twice a day. The blinking exercise is an exercise where the patient has to look at a far object for a period of 5 sec followed by blinking and keeping the eyelids closed for 5 sec. These two form one blinking exercise. Multiple repetitions of this cycle are carried out for five minutes in the morning and in the evening along with a few specific tests before and after six months of treatment.

All variables were assessed every two months for six months following treatment. The parameters observed and compared were as follows: change in uncorrected visual acuity (UCVA), change in refractive error in terms of SE, change in near point of convergence (NPC) and near point of accommodation (NPA), and change in Schirmer’s test, and tear film breakup time (TBUT).

Statistical analysis 

The statistical analysis was done using IBM SPSS version 23.0. All the continuous variables were expressed by mean±SD and categorical variables by frequency and percentage after the normality assumption check by Shapiro Wilk test. Descriptive statistics were calculated for all the parameters. For comparison between baseline and follow-up parameters, a paired t-test was used. For measuring the association between categorical variables, Chi-square test statistics were used. P-value <0.05 was considered as statistically significant.

## Results

In this study, there were 20 patients in the cases group and 18 in the control group. The maximum number of patients in the study (13 cases and 10 controls) were in the age group of 20-29 years. The sex-wise distribution of cases showed seven males and 13 females, while the control group had 12 males and six females. Table 1 shows the device usage, most patients in both groups used laptops (11 cases and 10 controls). Most patients used their device for 5-10 h (14 cases and 16 controls).

**Table 1:** Demographic and device usage details

Age (in years)	Cases (number)	Controls (number)
Less than 20	6	4
20-29	13	10
30-39	1	4
40 and more	0	0
Sex		
Male	7	12
Female	13	6
Occupation		
Professionals	11	9
Students	9	9
Type of devices		
Desktop	4	5
Laptop	11	10
Mobile phone	5	3
Duration of device Use		
Less than 5 h	3	0
5 to 10 h	14	16
10-15 h	3	2

Table 2 depicts the CVS-Q score, which significantly decreased in both cases and controls, with cases showing a greater reduction. The mean pre-treatment score for cases was 10.25±2.51, reduced to 5.30±1.34 post-treatment (p<0.001), indicating no CVS. Controls also showed a significant decrease from 9.39±2.12 to 7.78±1.66 (p<0.001), though minimal CVS features remained.

**Table 2:** Comparison of CVS score between case and control CVS-Q, computer vision syndrome questionnaire; CVS, computer vision syndrome

CVS-Q	Pre-treatment (mean±SD)	Post-treatment (mean±SD)	T-value	P-value
Cases	10.25±2.51	5.30±1.34	5.80	<0.001
Controls	9.39±2.12	7.78±1.67	4.40	<0.001

The mean visual acuity (VA) in cases improved significantly post-treatment, with the RE improving from 0.31±0.29 to 0.19±0.22 log MAR (p=0.002) and the left eye (LE) from 0.32±0.28 to 0.17±0.22 log MAR (p<0.001). Controls showed no significant mean VA changes. Refractive error (SE) in cases improved significantly, with the RE mean SE changing from -0.75±0.70 D to -0.5±-0.58 D (p 0.001) and the LE from -0.87±0.94 D to -0.56±0.58 D (p=0.021). Controls showed no significant SE change, indicating the treatment was effective in cases but not in controls (Table 3). This highlights the potential benefit of the treatment in improving both visual acuity and refractive error in affected individuals.

**Table 3:** Visual acuity (UCVA) and refractive error (SE)

Group	Eye	Parameter	Pre-treatment (mean±SD)	Post-treatment (mean±SD)	t-value	P-value
Cases	Right	Visual acuity (UCVA)	0.31±0.29	0.19±0.22	3.64	0.002
		Refractive error (SE)	-0.75±0.70	-0.50±0.58	-4.44	<0.001
	Left	Visual acuity (UCVA)	0.32±0.28	0.17±0.22	4.94	<0.001
		Refractive error (SE)	-0.87±0.94	-0.56±0.58	-2.52	0.021
Controls	Right	Visual acuity (UCVA)	0.27±0.25	0.22±0.23	1.92	0.072
		Refractive error (SE)	-0.96±1.14	-0.82±1.12	-1.82	0.086
	Left	Visual acuity (UCVA)	0.26±0.20	0.24±0.20	1.37	0.187
		Refractive error (SE)	-0.77±0.77	-0.67±0.72	-1.77	0.095

Table 4 showed the mean change in NPA before and after treatment between the two eyes in the cases was different and the values were 11.25±2.59 cm to 10.25±2.79 cm in RE and 11.20±2.56 cm to 10.40±2.82 cm in the LE. These were statistically significant with p-values of 0.027 in the RE and 0.080 in the LE, respectively.

**Table 4:** NPA and NPC NPA, near point of accommodation; NPC, near point of convergence

	Parameter	Eye	Pre-treatment (mean±SD)	Post-treatment (mean±SD)	t-value	P-value
Cases	NPA	Right	11.25±2.59	10.25±2.79	2.40	0.027
		Left	11.20±2.56	10.40±2.82	1.84	0.080
Controls		Right	11.89±1.90	11.39±1.54	2.15	0.046
		Left	11.56±1.54	11.61±1.61	-2.94	0.772
Cases	NPC		6.56±1.64	6.46±1.27	0.55	0.537
Controls			7.44±2.25	7.00±2.24	2.20	0.042

The mean NPA in the RE of the controls was 11.89±1.9 cm, which changed to 11.39±1.54 cm post-treatment. This was also of no statistical significance with a p-value of 0.046. In the LE, the mean NPA was 11.56±1.54 cm before treatment, which changed to 11.61±1.61 cm following treatment, accounting for no statistically significant change (p=0.772).

The mean NPC in cases changed from 6.56±1.64 cm to 6.46±1.27 cm post-therapy, showing no statistically significant difference. In controls, the NPC improved significantly from 7.44±2.25 cm to 7.0±2.24 cm (p=0.042).

Table 5 shows the dry eye examination consisting of the Schirmer strip test and TBUT in both the eyes of cases and controls assessed before and after the treatment The Schirmer’s test results showed no significant change in both groups pre- and post-treatment. In cases, the RE changed from 22.90±11.84 mm to 22.85±11.16 mm (p=0.963), and the LE from 23.50±12.03 mm to 23.61±10.61 mm (p=0.962). In controls, the RE changed from 24.17±5.31 mm to 24.39±4.80 mm (p=0.625), and the LE from 25.39±4.80 mm to 24.39±4.12 mm (p=0.132). This indicates that the treatment had no significant effect on tear production in either group over the six months. The lack of statistical significance suggests that other factors might influence tear production stability. The mean TBUT in cases improved significantly, with the RE increasing from 6.95±3.96 sec to 9.10±3.58 sec (p<0.001) and the LE from 7.35±3.40 sec to 9.60±3.36 sec (p<0.001). In controls, the RE TBUT decreased from 6.44±1.55 sec to 5.11±1.50 sec (p=0.010) and the LE from 6.33±1.61 sec to 4.28±1.45 sec (p=<0.001), indicating a significant reduction.

**Table 5:** Dry eye assessment - Schirmer’s test and TBUT TBUT, tear film breakup time

Group	Parameter	Eye	Pre-treatment (mean±SD)	Post-treatment (mean±SD)	t-value	P-value
Cases	Schirmer’s test	Right	22.90±11.84	22.85±11.16	0.06	0.963
		Left	23.50±12.03	23.61±10.61	-0.06	0.962
Controls		Right	24.17±5.31	24.39±4.80	-0.50	0.625
		Left	25.39±4.80	24.39±4.12	1.58	0.132
Cases	TBUT	Right	6.95±3.96	9.10±3.58	-3.12	<0.001
		Left	7.35±3.40	9.60±3.36	-3.65	<0.001
Controls		Right	6.44±1.55	5.11±1.50	4.78	0.010
		Left	6.33±1.61	4.28±1.45	7.33	<0.001

The grade of asthenopia (GOA) is measured by using an asthenopia questionnaire form, which changed in both groups pre- and post-treatment. The GOA changed from higher to lower grades with mild (8), moderate (10), severe (2) in the initial period to mild (11) and NIL had moderate or severe grades of asthenopia. This is of great significance with 45% of patients (cases) completely having NIL and 55% (11) having mild symptoms (Table 6).

**Table 6:** Asthenopia grades pre- and post-treatment

Group	Severity	Pre-treatment	Post-treatment	Chi-square	P-value
Cases	Mild	8	11	10.768	0.004
	Moderate	10	0		
	Severe	2	0		
Control	Mild	9	8	4.436	0.108
	Moderate	7	3		
	Severe	2	7		

## Discussion

Digital device use has become an indispensable tool in our everyday lives. COVID-19 increased computer usage, especially among adolescents and children, for professional and educational activities, causing a higher incidence of CVS. Focusing and refocusing constantly on images on the screen is necessary to provide clear near vision, which makes working with computers extremely taxing on the eyes. Extended working hours lead to both ocular and non-ocular ailments. The computer vision syndrome questionnaire (CVS-Q) assesses digital eye strain, including visual abnormalities, dry eye, irritation, and headaches. Effective management includes lifestyle modifications, exercise, and preventive measures.

In this study, most patients were under 30 years old (95% of cases, 77.78% of controls), similar to findings in other Indian and international studies [9-11]. The prevalence of CVS (68-69%) from many international data matches our study [12-16]. This could be due to this age group's extensive use of digital devices for professional and academic activities. The study had an equal number of males and females, with females predominating among the cases (65%) and males in the control group (66.67%). This aligns with other studies showing a higher prevalence of CVS in females [11,17,18]. The shift to online classes during COVID-19 has increased CVS prevalence among younger students. Also, in the present era of digitalization, none of the fields are spared from digital device use. Our study population was evenly spread between professionals (52.63%) and students (47.37%). Other studies have focused on specific groups, either students or professionals [19-21].

Of the total patients, about 67%, which is two-thirds of the population, used computers for 5-10 h. In the cases, 70% of the patients and in the controls, 88.89% of the patients used their devices for more than 5 hours. Thus, this variable remained comparable between the two groups, aiding in better analysis. However, it has been variably reported in other studies. In a study done on the African population, 85.8% of patients used their devices for longer hours (>7 h), and in another study from the same region, the majority of patients (55%) worked on digital devices for less than 5 h. This variability could be due to regional variation in occupation, awareness, and knowledge about the problem of CVS [22].

CVS assessment using the CVS-Q score helps to determine the severity of the condition, and monitoring it pre- and post-treatment guides the effectiveness of the treatment. The CVS-Q score drastically decreased among the cases, indicating significant improvement (p-value <0.001) with a phenomenal decrease in headaches and eye discomfort. This fact was probably a reflection of improvement in many parameters like changes in unaided vision, refractive error, dry eye, and orthoptic parameters.

Visual acuity significantly improved in the case group following blinking exercises. Almost all patients halted myopia progression, with many achieving emmetropia. The mean refractive error change in the RE (p<0.001) was significant. Controls showed no significant change in refractive error (p-values: RE, 0.086; LE, 0.095). This improvement is attributed to optimal visual function obtained by maintaining a stable and regular tear film through blinking exercises [22]. The refractive changes could be attributed to changes in the tear film. The irregular tear film significantly influences the human eye's optical quality. The likely explanation for the hazy vision is CVS-associated dry eye and irregular tear film-induced optical aberrations. Therefore, a consistently stable tear film with blinking exercises is critical to maintaining image quality for optimized visual functioning. However, the visual change due to this aspect is expected to be transient or ill-sustained.

Among cases, RE vision improved from 0.31±0.29 to 0.19±0.22 logMAR, and LE vision improved from 0.32±0.28 to 0.17±0.22 logMAR, both statistically significant despite regular screen use. Controls showed minimal change, not statistically significant (p-values: RE, 0.072; LE, 0.187). This suggests that extended blinking therapy leads to improved visual acuity and may increase retinal sensitivity, similar to amblyopia therapy [23,24].

The Generation R study links increased computer use in childhood to myopia progression, especially with a parental history of myopia, highlighting the need for interventions to reduce screen time and increase outdoor activities [24]. This underscores the importance of public health strategies to address myopia in childhood, while further research is needed to understand its impact in adulthood.

The NPA among cases showed significant improvement in the RE (p=0.027). Digital device use may cause tear film instability, leading to ocular fatigue and short TBUT. Symptoms of short TBUT are linked to tear film instability and vision impairment. Collier's study found no significant changes in accommodation due to computer use, though the mean NPC in cases was not statistically significant [25]. In controls, the NPC showed considerable improvement (p=0.042). Increased ciliary body tone does not exert the ciliary body thus leading to a lesser degree of asthenopia. The difference between both eyes could be due to the smaller sample size and duration of the study. A longer duration with the more bigger sample is needed to establish the findings.

A study showed that changes in NPA and NPC caused ocular discomfort and visual fatigue along with dry eye in CVS [26]. Regular breaks and proper ergonomics can mitigate these effects. Convergence exercises improve near-work tolerance. Changes in NPA in the RE may be due to blinking exercises, affecting intraocular and extraocular muscles, retina, and vision. This could be due to a small number of study samples. It needs a much larger duration with relatively larger samples along with longer follow-up to establish the uni- or bilaterality of the findings. 

Rosenfield et al. found no significant changes in accommodative or vergence facility post-computer use but noted a positive correlation between pre-task vergence facility and CVS symptoms like tired eyes and eyestrain [27]. This suggests potential ocular instability during computer use, highlighting the complexity of CVS and the need for further research. The study highlights the importance of regular breaks and proper screen ergonomics to prevent ocular pain and headaches caused by decreased convergence ability. This approach can help reduce CVS and improve screen time tolerance [24].

Despite normal Schirmer’s test results, TBUT was significantly abnormal, with p-values for both eyes being less than 0.001. Extended blinking improved TBUT from 6.95±3.96 sec to 9.10±3.58 sec in the RE and from 7.35±3.40 sec to 9.60±3.36 sec in the LE. This suggests blinking positively impacts tear film formation and reduces complications. The 20/20/20 rule significantly reduced dry eye symptoms (p=0.04) but limited overall CVS symptoms (p=0.38). Improvement of TBUT is definite in the case group. One of the major factors in this increase in TBUT is the blinking exercise. This has also been found in other studies. In the control group, there is definite deterioration of TBUT with increased dry eye with progressive CVS. 

Limitations of the study

This study had some limitations. The small sample size may affect the generalizability of the findings. The short-term follow-up was insufficient to observe long-term effects. Single-blinded randomization could introduce bias. Subgroup analysis based on profession was not performed. There was a lack of detailed evaluation of extraocular ergonomic factors and blinking patterns. Compliance with this home-based therapy is questionable and relies on self-reporting and adherence.

## Conclusions

There has been a drastic increase in computer device use globally, leading to a significant rise in CVS. This condition often causes ocular surface irritation, visual problems, and dry eyes. Blinking therapy can effectively reduce CVS symptoms, improve visual acuity, and reduce refractive errors. While Schirmer's test scores remained low, most cases saw an increase in TBUT, indicating reduced dryness. The combined effects of conventional treatment and blinking exercises significantly decreased CVS-Q scores, enhancing visual clarity and computer screen tolerance. Extended blinking therapy offers a holistic solution for adults and children who use computers for extended periods.
